# Recent Progress in Nanomaterial-Based Surface-Enhanced Raman Spectroscopy for Food Safety Detection

**DOI:** 10.3390/nano14211750

**Published:** 2024-10-31

**Authors:** Hagar S. Bahlol, Jiawen Li, Jiamin Deng, Mohamed F. Foda, Heyou Han

**Affiliations:** 1 National Key Laboratory of Agricultural Microbiology, College of Life Science and Technology, College of Chemistry, Huazhong Agricultural University, Wuhan 430070, China; hager.shendy@fagr.bu.edu.eg (H.S.B.); jiawenli@webmail.hzau.edu.cn (J.L.); dengjiamin2018@webmail.hzau.edu.cn (J.D.); 2 Department of Biochemistry, Faculty of Agriculture, Benha University, Moshtohor, Toukh 13736, Egypt; 3 National Key Laboratory of Crop Genetic Improvement, College of Life Science and Technology, Hubei Hongshan Laboratory, Huazhong Agricultural University, Wuhan 430070, China

**Keywords:** SERS, biosensors, molecular fingerprint, foodborne pathogens, food packaging

## Abstract

Food safety has recently become a widespread concern among consumers. Surface-enhanced Raman scattering (SERS) is a rapidly developing novel spectroscopic analysis technique with high sensitivity, an ability to provide molecular fingerprint spectra, and resistance to photobleaching, offering broad application prospects in rapid trace detection. With the interdisciplinary development of nanomaterials and biotechnology, the detection performance of SERS biosensors has improved significantly. This review describes the advantages of nanomaterial-based SERS detection technology and SERS’s latest applications in the detection of biological and chemical contaminants, the identification of foodborne pathogens, the authentication and quality control of food, and the safety assessment of food packaging materials. Finally, the challenges and prospects of constructing and applying nanomaterial-based SERS sensing platforms in the field of food safety detection are discussed with the aim of early detection and ultimate control of foodborne diseases.

## 1. Introduction

### 1.1. The Necessity and Importance of Rapid Food Safety Detection Methods

Food safety is a critical aspect of safeguarding public health and ensuring consumer wellbeing, particularly in the context of the increasingly interconnected global food trade and supply chains. The growing recognition of chemical pollution, microbial contamination, and adulteration in food has emphasized the importance of implementing effective safety measures [1]. Consequently, rapid detection methods are paramount in this scenario. Often limited by their labor-intensive and time-consuming nature, traditional detection technologies cannot meet current demands. Hence, there is an urgent need to develop rapid, convenient, and accurate analytical and detection techniques. These innovative methods represent a future trend in food safety as they address the limitations of conventional approaches and enable timely interventions and effective risk management [2].

Rapid detection methods play a pivotal role in upholding food safety standards by enabling swift identification of biological and chemical contaminants in food. These methods encompass a range of techniques, including culture and colony counting, immunology-based methods, polymerase chain reaction, and serological approaches [3,4]. By providing rapid results, they can contribute to the prevention of outbreaks, curb the spread of contamination, and mitigate the economic impact on food producers, distributors, and retailers. Furthermore, rapid detection methods contribute to regulatory compliance, quality assurance, enhanced traceability, and advancements in detection technologies [5,6]. Their multifaceted benefits underline their significance in ensuring the safety and integrity of the global food supply. The significance of food safety cannot be overstated and the need for rapid detection methods is vital for effective food safety measures. Owing to their ability to identify contaminants swiftly, these methods offer invaluable support for maintaining the safety of food products. By embracing rapid detection techniques, food industry stakeholders can enhance their ability to respond to emerging threats, safeguard public health and build consumer trust.

### 1.2. History and Discovery of Surface-Enhanced Raman Spectroscopy (SERS)

SERS is a powerful analytical technique that enhances Raman scattering of molecules adsorbed on rough metal surfaces or nanostructures. Its history began in the early 1970s and was marked by significant discoveries and advancements.

The SERS phenomenon was discovered and first observed accidentally in 1974 by Martin Fleischmann et al. during experiments involving the Raman scattering of pyridine on roughened silver electrodes. They noted an unexpected increase in the intensity of the Raman signal, which was initially attributed to the surface area effect. This discovery laid the groundwork for further investigation into the mechanisms underlying this enhancement. In 1977, two independent research groups, led respectively by Jeanmaire and Richard Van Duyne and by Albrecht and Creighton, explored the enhancement factors and proposed values between 105 and 106. They suggested that the enhancement could not be solely explained by the concentration effects, leading to the introduction of electromagnetic and charge transfer mechanisms as explanations for the observed phenomena [7,8,9]. The concept of plasmon excitation was also highlighted during this period, linking SERS to the excitation of surface plasmons in nanostructured metals, a connection elaborated on by Moskovits in subsequent years.

Mechanisms of SERS enhancement is primarily attributed to two mechanisms:

1. Electromagnetic enhancement: This occurs because of localized surface plasmon resonance (LSPR) in metallic nanostructures, which amplifies the electric field near the surface, thereby increasing the Raman scattering cross-section of the molecules present.
2. Charge transfer mechanism: This involves the transfer of charge between the adsorbate (the molecule being studied) and the metal surface, which can also enhance the Raman signal [10].

SERS has evolved significantly since its discovery and has become a field of extensive research, with thousands of publications exploring its theoretical foundations, substrate designs, and various applications. SERS is now recognized for its high sensitivity and capability to detect single molecules, making it invaluable in fields such as biology, medicine, environmental monitoring, and materials science [10,11].

Recent advancements have included the development of novel SERS substrates, such as plasmonic paper and other nanostructured materials, which have improved the accessibility and cost-effectiveness of SERS measurements. These innovations have enabled applications in clinical diagnostics, including the early detection of cancer biomarkers and other medical diagnostics [12,13,14].

### 1.3. Overview of Surface-Enhanced Raman Spectroscopy (SERS) Technique

SERS is a highly versatile and sensitive analytical technique that combines principles of Raman spectroscopy with nanotechnology to enhance the detection and characterization of molecules. It has gained significant attention in various research fields and applications owing to its ability to provide detailed molecular information with exceptional sensitivity and spatial resolution [15].

**Figure 1:**
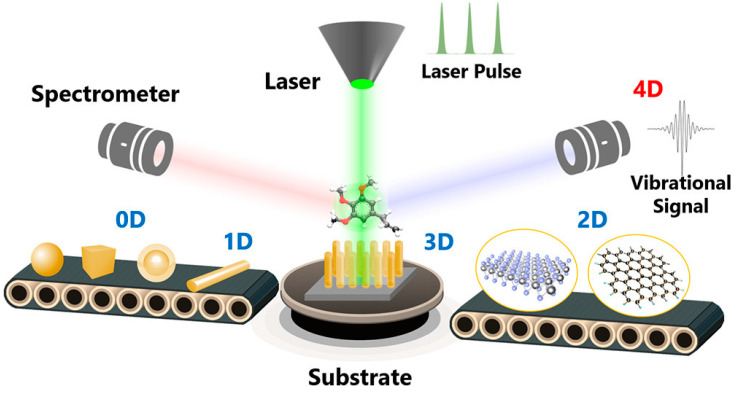
Illustration of multidimensional spectroscopy and nanomaterial dimensions: Schematic of multidimensional spectroscopy setup. A laser generates pulses to excite a sample on a substrate, producing vibrational signals that are subsequently captured by a spectrometer. Nanomaterials, categorized by their dimensions, include 0D spherical and cubic nanoparticles, 1D nanotubes and nanorods, 2D graphene and other layered materials, and 3D nanostructured arrays. Reprinted with permission from ACS Mater. Au 2022, 2, 5, 552–557 [15].

As shown in Figure 1, SERS relies on the interaction between the incident laser light and metallic nanostructures with different dimensional levels (0D, 1D, 2D, 3D, and 4D) implemented on a substrate, typically gold or silver nanoparticles, to generate surface plasmon resonance [16]. This interaction significantly enhances the Raman scattering signal, enabling the detection of trace amounts of analytes. This enhancement arises from two main effects: the electromagnetic field and chemical enhancements. Electromagnetic field enhancement is achieved by amplifying the Raman signals of nearby molecules by several orders of magnitude, whereas the chemical enhancement effect results from charge transfer between the analyte molecules and metal surface [17].

The unique properties of SERS can be attributed to its ability to precisely control the physical and chemical properties of metallic nanostructures. Various fabrication techniques, including colloidal synthesis, lithography, and self-assembly, can be used to create well-defined nanostructures with tailored sizes, shapes, and compositions. These nanostructures can be further functionalized with specific molecular probes or recognition elements, enabling the selective detection of target analytes in complex samples [18,19,20].

The applications of SERS span numerous scientific disciplines. In chemistry, SERS is used to analyze molecular structures, identify chemical reactions, and monitor catalytic processes. In biology and medicine, SERS enables the detection and imaging of biomolecules, such as DNA, proteins, and metabolites, contributing to advancements in disease diagnostics and therapeutics [21]. Environmental science benefits from SERS by detecting and monitoring pollutants, contaminants, and hazardous substances in various samples [22,23,24]. SERS has applications in forensic analysis, food safety, art conservation, and several other fields.

With the ongoing advancements in nanotechnology and spectroscopic instrumentation, SERS continues to evolve and holds great promise for future innovations. Its unique capabilities for the sensitive and selective detection of molecules make it a valuable tool for research, analysis, and various practical applications. The combination of enhanced sensitivity, spatial resolution, and ability to tailor the properties of metallic nanostructures makes SERS an indispensable technique for scientists and researchers working in diverse scientific domains.

The size and shape of the nanoparticles also significantly affect SERS intensity and spectrum. As the nanoparticle size increases, so too does the total Raman intensity, and the lower-energy Raman modes became dominant. This behavior is due to the overlap between the plasmonic modes of the dimer structure and the Raman spectrum. Larger nanoparticles cause a redshift in the plasmonic dipolar mode, altering its overlap with the Raman spectrum and providing external control over emission properties [25].

### 1.4. Comparison of SERS with Commonly Used Detection Methods in Food Safety

Food safety testing plays a crucial role in public health, and various conventional analytical methods, such as enzyme-linked immunosorbent assay (ELISA), polymerase chain reaction (PCR), and high-performance liquid chromatography (HPLC), are commonly utilized to detect food contaminants. Although these techniques offer substantial reliability, they also have limitations in terms of efficiency, cost, and ease of use.

ELISA, which is widely recognized for its high specificity and sensitivity, often requires substantial sample volumes and is limited by its time-intensive nature and the need for trained personnel to perform the assay [26]. Furthermore, ELISA is not suitable for real-time monitoring, which is a growing requirement for food safety assessments. Similarly, PCR is highly sensitive in detecting specific nucleic acids but is constrained by its relatively high cost due to the necessity of specialized equipment and reagents. PCR assays are prone to contamination and require amplification steps that introduce delays, making PCR unsuitable for rapid on-site applications [27]. HPLC, another widely adopted method for detecting chemical contaminants, is known for its high precision and accuracy. However, the requirement for expensive solvents, labor-intensive procedures, and lengthy analysis time limits its applicability for high-throughput, real-time detection [28], and the complex sample preparation required for HPLC adds to the overall cost and time burden of the method.

By contrast, SERS offers distinct advantages over traditional methods. SERS enables the highly sensitive and rapid detection of analytes with minimal sample preparation, providing real-time analysis at the molecular level. Unlike ELISA, PCR, and HPLC, SERS can detect trace levels of contaminants without using complex reagents or amplification processes. This reduces operational costs and makes the method highly adaptable to diverse food safety applications [29]. Furthermore, SERS allows the detection of a wide range of contaminants by providing a unique molecular fingerprint of the analytes, making it a versatile and cost-effective alternative to conventional methods [30].

By overcoming the limitations of time-consuming sample preparation, the high cost, and the complex operational demands associated with ELISA, PCR, and HPLC, SERS is a highly promising alternative for food safety testing. Its capacity for rapid, sensitive, and real-time detection underscores its potential to revolutionize the current landscape of food safety analysis.

## 2. Principles of SERS

### 2.1. Why Has SERS Emerged as a Powerful Tool to Rapidly Detect Food Safety Issues?

Surface-enhanced Raman spectroscopy has emerged as a powerful tool for rapidly detecting food safety issues, owing to its unique capabilities and numerous advantages. This advanced spectroscopic technique has revolutionized food analysis by providing the sensitive, selective, and rapid detection of various contaminants, toxins, and adulterants in food samples [31].

One of the primary reasons for the effectiveness of SERS in food safety analyses is its exceptional sensitivity. SERS can detect trace amounts of analytes even at concentrations as low as parts per billion. This level of sensitivity is crucial for identifying potential hazards in food, as contaminants and toxins can pose significant health risks even at very low concentrations [32]. By amplifying Raman signals through plasmonic enhancement, SERS enables the detection of minute quantities of harmful substances, allowing for the early intervention and prevention of foodborne illnesses.

Another key advantage of SERS is its rapid detection capability. Unlike traditional analytical methods that require time-consuming sample preparation and complex laboratory procedures, SERS provides real-time results. It offers rapid and on-site analysis, eliminates the need for sample transportation, and reduces the decision-making time. The ability to quickly detect and identify contaminants in food samples enables timely intervention to prevent the distribution of unsafe products, protect public health, and ensure the safety of the food supply chain [33,34].

Furthermore, SERS is highly specific and provides the selective detection of target analytes. This specificity is crucial for food safety analysis, in which the presence of specific contaminants or adulterants must be accurately identified. SERS can be used to distinguish different chemical species based on their unique Raman spectra, thereby enabling the identification and quantification of specific analytes in complex food matrices. This specificity ensures reliable and accurate detection, thereby reducing the number of false positives and negatives in food safety assessment.

Moreover, SERS offers a non-destructive and non-invasive approach to food analysis. This method requires minimal sample preparation, preserves the integrity of food samples, and minimizes waste. The non-destructive nature of SERS allows the analysis of multiple samples without compromising their quality or usability. This aspect is particularly beneficial in quality control and regulatory inspections where large-scale screening of food products is necessary [35,36,37].

SERS has emerged as a powerful tool for rapidly detecting food safety issues because of its extraordinary sensitivity, rapid detection capability, selectivity, and non-destructive nature. As the importance of food safety continues to increase, the application of SERS in the food industry has offered significant advantages in terms of early detection, accurate identification, and timely intervention. Continued advancement and integration of SERS technology into food safety protocols will enhance our ability to ensure the safety and quality of the food we consume, safeguard public health, and strengthen consumer confidence.

### 2.2. Raman Scattering: Explanation of the Phenomenon and Its Significance

Raman scattering is a spectroscopic phenomenon that involves the inelastic scattering of light by molecules, resulting in a shift in the energy of the scattered photons. This effect was discovered by Indian physicist Sir C. V. Raman in 1928 and has since become an essential tool in various fields of science, including chemistry, physics, materials science, and biology [38,39].

Raman scattering occurs when a photon interacts with a molecule and imparts or absorbs energy from molecular vibrations or rotations. Consequently, the scattered photons have different energies than the incident photons, resulting in a characteristic Raman spectrum. The energy shift of scattered photons corresponds to the vibrational or rotational energy levels of the molecule, providing valuable information regarding its molecular structure and chemical composition.

Raman scattering is significant for several reasons. Firstly, it allows for the identification and characterization of chemical substances. The Raman spectrum obtained from a sample serves as a unique molecular fingerprint that enables identification of specific compounds. By comparing the Raman spectrum of an unknown sample with reference spectra, researchers can determine the composition and structure of a material, even in complex mixtures [40].

Secondly, Raman scattering provides insights into molecular vibrations and interactions. The intensity and pattern of the Raman peaks in the spectrum provide information about the vibrational modes of the molecules, such as the stretching, bending, and twisting motions. These data can be used to study molecular conformation, intermolecular forces, and chemical bonding within a substance. Raman spectroscopy is particularly useful for studying solids, liquids, and gases, as it can be applied to a wide range of sample types.

Moreover, Raman spectroscopy offers advantages in terms of non-destructive and non-invasive analysis. It requires minimal sample preparation, often allowing for the direct analysis of solids, liquids, and gases, without the need for complex sample handling. This feature makes Raman spectroscopy suitable for in situ and real-time measurements, preserving the integrity of the sample and facilitating in-depth analysis of delicate or valuable materials [41].

Raman scattering has also been applied in various scientific disciplines. In chemistry, it is used to analyze organic and inorganic compounds, monitor chemical reactions, and study molecular dynamics. In materials science, Raman spectroscopy provides valuable information regarding the structure, composition, and defects of materials, including semiconductors, polymers, and nanomaterials. In biology and medicine, Raman spectroscopy is utilized for cellular imaging, disease diagnosis, and drug development, offering non-invasive and label-free analysis of biological samples [42].

Raman scattering is a fundamental phenomenon that enables the analysis of molecular structures, vibrations, and interactions. Its significance lies in its ability to provide unique molecular fingerprints, insights into molecular dynamics, non-destructive scrutiny, and wide-ranging applications in various scientific fields. Raman spectroscopy continues to be a valuable tool for researchers and scientists in their quest to understand the fundamental properties of matter and advance knowledge in diverse disciplines.

### 2.3. Enhancement Mechanisms: Introduction to Plasmonic Nanoparticles and Their Role in SERS

Enhancement mechanisms play a crucial role in the effectiveness of SERS, a powerful technique for enhancing the Raman signals of molecules. One of the key components of SERS is the use of plasmonic nanoparticles, which enhance the Raman signal through various mechanisms [43]. In this review, we introduce plasmonic nanoparticles and discuss their role in SERS.

Plasmonic nanoparticles are metallic nanoparticles typically composed of materials such as gold (Au) or silver (Ag), which exhibit localized surface plasmon resonance (LSPR). LSPR is the collective oscillation of conduction electrons in response to incident light, resulting in the enhancement of the electric field near the nanoparticle surface. This enhanced electric field interacts with nearby molecules, leading to the amplification of the Raman scattering signal.

Nanoparticle-based surface-enhanced Raman scattering is a powerful analytical technique, and its efficacy is largely determined by the size, shape, and surface functionalization of nanoparticles, which influence localized surface plasmon resonance (LSPR) and the formation of electromagnetic “hotspots.” The size of the nanoparticles plays a crucial role in enhancing the SERS signals. Nanoparticles between 10 and 100 nm typically offer optimal enhancement, with smaller particles providing a higher surface-to-volume ratio, leading to greater analyte interactions. However, larger particles may result in a redshift in the plasmon resonance, altering the SERS response [44].

The shape also significantly affects the SERS performance. Nanoparticles with anisotropic shapes, such as rods, cubes, or stars, create stronger electromagnetic “hotspots” compared with spherical particles. These sharp-edged shapes can generate more intense localized plasmonic fields, resulting in enhanced signals. The enhancement order typically follows nanospheres < nanosphere aggregates < nanotriangles < nanostars [45,46,47].

Finally, surface functionalization is crucial to improve the selectivity of SERS detection. Modifying nanoparticle surfaces with specific ligands, such as thiols or antibodies, enhances analyte adsorption, improving sensitivity and selectivity.

One of the primary enhancement mechanisms associated with plasmonic nanoparticles is electromagnetic (EM) enhancement. When a plasmonic nanoparticle is illuminated with light that matches its LSPR frequency, the electric field in the vicinity of the nanoparticle intensifies significantly. This enhanced electric field interacts with target molecules, increasing their Raman scattering signals by several orders of magnitude. The EM enhancement mechanism relies on the strong interaction between the localized surface plasmons of the nanoparticles and the incident light.

Chemical enhancement is another enhancement mechanism. In this mechanism, plasmonic nanoparticles provide a favorable environment for chemical interactions between the target molecules and nanoparticle surfaces. The presence of nanoparticles alters molecular properties, such as the electronic structure and charge distribution, leading to an enhanced Raman signal. Chemical enhancement can arise from charge transfer processes, surface-enhanced charge transfer (SECT), or changes in molecular polarizability [48].

In addition, plasmonic nanoparticles contribute to this enhancement through hotspots. Hotspots are regions of highly enhanced electric fields that occur in the gaps or junctions between closely spaced nanoparticles. These regions exhibit even stronger field enhancements than those of the individual nanoparticles, leading to highly amplified Raman signals. The precise control of nanoparticle size, shape, and arrangement allows for the manipulation and optimization of hotspots, further enhancing the SERS signal [14,49].

Thus, plasmonic nanoparticles play a pivotal role in SERS by providing enhancement mechanisms that amplify the Raman signals of molecules. The electromagnetic enhancement, chemical enhancement, and hotspot effects generated by plasmonic nanoparticles contribute to the significant enhancement observed in the SERS experiments. Understanding these enhancement mechanisms and the role of plasmonic nanoparticles is crucial for designing and optimizing SERS substrates, leading to improved sensitivity and detection capabilities in a wide range of applications, including chemical sensing, biomedical diagnostics, and environmental monitoring.

### 2.4. Signal Enhancement: Description of the SERS Effect and Its Amplification Mechanisms

Signal enhancement is a fundamental aspect of SERS, a powerful technique used to amplify the Raman scattering signals of molecules. The SERS effect arises from the interaction between molecules and specially designed surfaces that are typically coated with plasmonic nanoparticles. This interaction leads to significant amplification of the Raman signal, enabling highly sensitive detection and characterization of molecules [50].

This SERS effect can be attributed to several amplification mechanisms. One of the key mechanisms is electromagnetic (EM) enhancement, which results from the interaction between incident light and plasmonic nanoparticles. Plasmonic nanoparticles exhibit localized surface plasmon resonance (LSPR), a phenomenon in which the collective oscillation of conduction electrons is induced by the incident light. This LSPR generates intense localized electric fields around the nanoparticles, significantly enhancing the Raman scattering signal of the nearby molecules. The EM enhancement factor can reach values of 10^6^ to 10^10^, enabling the detection of even single molecules [51].

Another amplification mechanism in SERS is chemical enhancement. This mechanism relies on chemical interactions between the molecules and the SERS substrate. The presence of plasmonic nanoparticles modifies the electronic structure and charge distribution of the molecules, leading to an increased Raman scattering signal. Chemical enhancement can arise from various processes, such as charge transfer between the molecule and the nanoparticle surface or modifications in molecular polarizability. The exact nature of the chemical enhancement mechanism depends on the specific molecule–substrate interactions and can vary for different systems [52].

Furthermore, the spatial arrangement of plasmonic nanoparticles plays a crucial role in SERS signal enhancement. When nanoparticles are closely spaced, such as in aggregated or nanoparticle-assembled structures, regions of highly enhanced electric fields, known as “hotspots,” are formed. Hotspots occur at nanoscale gaps or junctions between nanoparticles where the electric fields are significantly intensified. These hotspots can amplify the Raman signal, further boosting the sensitivity of SERS measurements [53].

The combination of electromagnetic enhancement, chemical enhancement, and hotspot effects contributes to the overall signal enhancement observed in SERS. These amplification mechanisms enable the detection of trace amounts of molecules and enhance the sensitivity and selectivity of the Raman spectroscopy. The precise control of nanoparticle properties, such as size, shape, and composition, allows for the manipulation and optimization of these amplification mechanisms, leading to improved SERS performance [54].

In summary, the SERS effect is characterized by remarkable amplification of the Raman scattering signal through various mechanisms. The electromagnetic enhancement, chemical enhancement, and hotspot effects offered by plasmonic nanoparticles collectively contribute to significant signal enhancement in SERS. Understanding these amplification mechanisms is crucial for designing and developing highly sensitive and selective SERS substrates, providing opportunities for applications in diverse fields including chemical analysis, medical diagnostics, ecological surveillance, and the appraisal of food safety.

## 3. Advantages of SERS in Food Safety Detection

### 3.1. High Sensitivity and Selectivity: Detecting Trace Analytes with Precision

**Figure 2:**
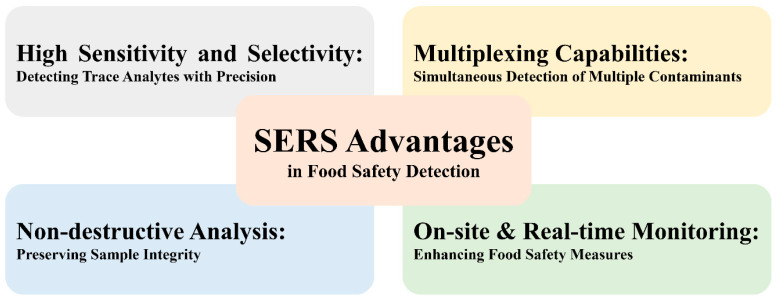
Overall advantages of SERS in food safety detection.

High sensitivity and selectivity are key advantages of SERS for the precise detection of trace analytes. SERS can identify and quantify extremely low concentrations of analytes even at the single-molecule level, as presented in Table 1. This high sensitivity is attributed to signal enhancement mechanisms inherent to SERS, such as electromagnetic enhancement, chemical enhancement, and hotspot effects. Figure 2 shows the overall advantages of SERS for food safety detection.

The electromagnetic enhancement in SERS generates intense localized electric fields around the plasmonic nanoparticles, leading to the amplification of the Raman scattering signal. This allows for the detection of analytes at much lower concentrations than conventional Raman spectroscopy. The sensitivity of SERS can be further improved by optimizing the size, shape, and composition of the nanoparticles, as well as their spatial arrangement, to create hotspots [50].

In addition to its sensitivity, SERS offers excellent selectivity in the detection of trace analytes. This selectivity arises from the characteristic vibrational fingerprints of the molecules, which provide unique Raman spectra for the different analytes. By analyzing specific Raman spectral features, it is possible to differentiate and identify different compounds within complex mixtures. This enables the precise detection of target analytes, even in the presence of interfering substances.

SERS selectivity can be enhanced using various strategies. One approach is to functionalize the SERS substrate with specific capture molecules or ligands that can selectively bind to target analytes. This promotes selective enrichment of the analytes of interest, improving their detection and reducing interference from other components in the sample. Additionally, advanced data analysis techniques, such as chemometric analysis and machine learning algorithms can be employed to enhance selectivity by identifying patterns and correlations in complex spectral data [55,56].

**Table 1:** Detection limit analysis, sensitivity, and specificity for various contaminants using nanomaterial-based sensors.

Contaminant Type	Contaminant	Detection Limit (µg/L)	Sensitivity (Slope)	Specificity (%)	Ref.
**Pesticides**	2,4-Dichlorophenoxyacetic acid (2,4-D)	0.11	0.001–100	89.73–100.27	[57]
	Methomyl	0.5	0.90	95	[58]
**Heavy Metals**	Lead	1.0	0.85	97	[59]
	Cadmium	0.5	1.0	96	[60]
**Pathogens**	Salmonella	10	1.5	99	[61]
	*E. coli*	5	1.3	98	[62]
**Toxins**	Aflatoxin	0.2	1.1	97	[63]
	Mycotoxin	0.3	1.0	95	[64]

The combination of the high sensitivity and selectivity of SERS makes it a powerful tool for the detection of trace analytes with high precision. This has significant implications in various fields including environmental monitoring, food safety, pharmaceutical analysis, and forensic science. In food safety, SERS can detect and quantify contaminants, such as pesticides, heavy metals, and foodborne pathogens, at very low concentrations, ensuring the safety and quality of food products, which we will introduce in this review.

Overall, the high sensitivity and selectivity of SERS enable precise detection of trace analytes. SERS offers a robust analytical technique for a wide range of applications by leveraging signal enhancement mechanisms and unique vibrational fingerprints of molecules. Its ability to detect and identify analytes at ultralow concentrations opens new possibilities for research, diagnostics, and monitoring, ultimately contributing to improved safety and quality control in various industries [65,66,67].

### 3.2. Multiplexing Capabilities: Simultaneous Detection of Multiple Contaminants

Multiplexing capability is the ability of a technique to simultaneously detect and identify multiple contaminants or analytes in a single measurement. Multiplexing has emerged as a powerful feature that expands SERS analytical capacity. SERS enables the simultaneous detection and identification of multiple contaminants by exploiting the unique vibrational fingerprints of different molecules, providing valuable information regarding complex samples in a time-efficient manner.

The multiplexing capabilities of SERS stem from the distinct Raman spectral signatures exhibited by the different analytes. Each analyte possesses a characteristic Raman scattering spectrum, resulting from the vibrational modes of its constituent molecules. These vibrational modes give rise to specific Raman peaks or bands, which can be assigned to different chemical species. By collecting and analyzing the Raman spectra of a sample using SERS, multiple contaminants can be identified based on their unique spectral patterns [68,69].

Several strategies can be employed to achieve multiplexing in SERS systems. One such approach involves the use of plasmonic nanomaterials with various properties. Selective binding and enrichment of different analytes can be achieved by functionalizing each nanoparticle type with specific capture ligands or molecules. Consequently, when these nanoparticles are mixed in a sample and analyzed using SERS, the distinct Raman spectra of the analytes can be simultaneously captured, enabling multiplexed detection [70].

Another strategy for multiplexing SERS involves using spatially encoded substrates. These substrates contain spatially separated regions, each designed to interact with a specific analyte. By applying the sample to such a substrate and performing SERS measurements, Raman spectra from each region can be obtained simultaneously. This approach allows the detection of multiple analytes in a single experiment, thereby reducing the analysis time and increasing throughput [71,72].

The ability to perform multiplexed detection using SERS has significant implications for various fields. For instance, in ecological monitoring, the simultaneous detection of multiple contaminants in water or soil samples using SERS can provide comprehensive information on pollution levels and identify the sources of contamination. In biomedical applications, SERS multiplexing can enable the detection of multiple disease biomarkers in a single patient sample, thereby facilitating rapid and accurate diagnoses [73,74].

Overall, the multiplexing capabilities of SERS offer valuable advantages for analytical chemistry. By harnessing the unique spectral signatures of different analytes, SERS enables simultaneous detection and identification of multiple contaminants or analytes in complex samples. This capability enhances the efficiency and throughput of the analysis, making SERS a versatile and powerful tool for a wide range of applications, including environmental monitoring, biomedical research, and food safety assessment.

### 3.3. Non-Destructive Analysis: Preserving Sample Integrity

Non-destructive analysis is a fundamental aspect of scientific investigation, particularly in the field of analytical chemistry, as it preserves the sample integrity during the measurement process. In the context of SERS, non-destructive analysis has emerged as a notable advantage, enabling the examination of samples without causing irreversible changes or damage.

SERS offers a non-destructive approach to sample analysis utilizing Raman scattering. Unlike other analytical techniques, which may require sample preparation or alteration, SERS measurements can be performed directly on a sample of interest, requiring minimal or no sample manipulation. This characteristic is particularly advantageous when studying sensitive or valuable samples that must be preserved for further analysis or for archival purposes.

The non-destructive nature of SERS is attributed to the low power requirements of Raman spectroscopy. Raman scattering occurs when incident photons interact with the sample’s molecular vibrations, resulting in the emission of scattered photons with different energies. The intensity and frequency of these scattered photons provide information on the chemical composition and molecular structure of the sample. In SERS, plasmonic nanoparticles are employed to enhance the Raman signal, amplifying the sensitivity of the technique while maintaining the non-destructive nature of the measurement [75,76].

By using SERS, researchers can investigate a wide range of samples without compromising their integrity. This includes the analysis of delicate biological specimens such as proteins, cells, and tissues, where maintaining the native state of the sample is crucial for accurate characterization. Non-destructive analysis with SERS also finds application in the study of historical artifacts, where preserving the integrity of cultural heritage objects is of utmost importance [77,78,79].

The non-destructive nature of SERS also extends to the analysis of complex matrices, such as food and environmental samples. With SERS, it is possible to analyze these samples without altering their chemical composition, ensuring the accurate detection of contaminants or trace analytes, while minimizing the risk of false positives or false negatives. This capability makes SERS an invaluable tool for food safety assessment, quality control, and environmental monitoring, for which preservation of sample integrity is paramount [80,81,82].

In conclusion, the non-destructive analysis offered by SERS allows the examination of samples without causing irreversible changes or damage. By harnessing the principle of Raman scattering and utilizing plasmonic nanoparticles, SERS enables the sensitive detection and characterization of various samples while preserving their integrity. This feature makes SERS a versatile and valuable tool in scientific research, offering a non-destructive analysis of delicate biological specimens, historical artifacts, and complex matrices.

### 3.4. On-Site and Real-Time Monitoring: Enhancing Food Safety Measures

On-site and real-time monitoring have emerged as crucial aspects for ensuring food safety and enhancing overall quality control measures. In analytical chemistry, SERS has garnered significant attention as a powerful technique for on-site and real-time monitoring, enabling rapid and reliable detection of food contaminants and ensuring the safety of food products throughout the supply chain.

Portability and user-friendliness are significant advantages of employing SERS for on-site monitoring. SERS instruments can be designed in compact and handheld formats, allowing their direct deployment at food production, processing, or distribution sites. This enables the real-time analysis of food samples without the need for sample transportation to a centralized laboratory, thereby minimizing delays in obtaining critical information and facilitating immediate corrective actions [83,84].

The high sensitivity and specificity of SERS contribute to its efficacy for on-site monitoring. This technique exhibits exceptional sensitivity, which enables the detection of trace levels of contaminants and adulterants in food samples. This sensitivity is further enhanced by plasmonic nanoparticles used in SERS, which amplify the Raman signal and facilitate analyte detection at extremely low concentrations. Thus, SERS can identify potential hazards or contaminants in real time, ensuring prompt intervention and preventing the distribution of unsafe food products. Moreover, the rapid analysis capabilities of SERS make it well suited for on-site monitoring. Traditional analytical methods often require extensive sample preparation, complex instrumentation, and time-consuming procedures. In contrast, SERS enables direct analysis of food samples with minimal sample preparation, offering near-instantaneous results. This real-time analysis capability not only expedites the detection of contaminants but also facilitates immediate decision making regarding the safety and quality of food products.

On-site and real-time monitoring provided by SERS enhances food safety measures by enabling proactive interventions. By swiftly detecting and identifying contaminants or adulterants, SERS empowers food producers, processors, and regulatory authorities to implement timely interventions such as halting production, recalling products, or implementing corrective actions to prevent further contamination. This proactive approach ensures the protection of public health and enhances consumer confidence in the safety and quality of food products [85,86,87].

In conclusion, the on-site and real-time monitoring facilitated by SERS plays a crucial role in enhancing food safety measures. The portability, sensitivity, specificity, and rapid analytical capabilities of SERS enable the immediate detection and identification of contaminants or adulterants in food samples, allowing for prompt intervention and preventive actions. By providing real-time analytical information, SERS empowers food industry stakeholders to ensure the safety and quality of food products, protect public health, and maintain consumer trust.

## 4. Applications of SERS in Food Safety Detection

### 4.1. Detection of Biological and Chemical Contaminants

#### 4.1.1. Pesticides and Herbicides

The detection of biological and chemical contaminants, specifically pesticides and herbicides, in food samples is of utmost importance for ensuring food safety and public health. SERS has emerged as a promising analytical technique, with significant potential in this field. This section focuses on the application of SERS for the detection and quantification of pesticides and herbicides and highlights its advantages and contributions to the field [88].

Pesticides and herbicides are widely used in agriculture to protect crops from pests, diseases, and weeds. However, the excessive and improper use of these chemicals can lead to their accumulation in food products, posing potential health risks to consumers. Traditional methods for detecting pesticides and herbicides often involve time-consuming sample preparation and complex analytical procedures. SERS is a rapid and sensitive alternative for the detection of contaminants in food samples.

**Figure 3:**
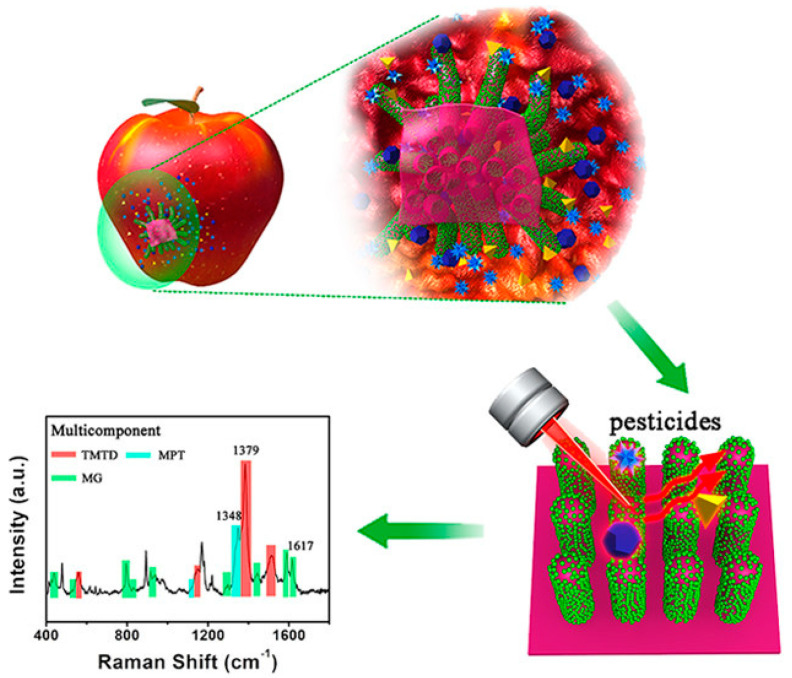
Detection of pesticides on fruit using Raman spectroscopy. Illustration of pesticide detection in apples using Raman spectroscopy. The inset shows pesticide molecules on the apple surface. A laser from the Raman spectrometer was used to target the surface to produce the Raman spectrum. The graph indicates the presence of pesticides (TMTD, MPT, and MG) with distinct peaks at specific Raman shifts (e.g., 1348, 1379, and 1617 cm^−1^), enabling the precise identification and quantification of pesticide residues. Reprinted with permission from Analytical Chemistry, 2017, 89(4), 2424–2431 [180].

One of the primary benefits of employing SERS for pesticide and herbicide detection is its exceptional sensitivity [89]. The enhancement of Raman signals through plasmonic effects enables the detection of trace amounts of these contaminants even in complex matrices. Selective capture and detection of target pesticides and herbicides can be achieved by functionalizing SERS-active substrates with specific receptors, such as aptamers or antibodies. This allows for the quantification of these contaminants at low concentrations, surpassing the limitations of conventional detection methods. Han et al. (2017) have developed a novel nanopillar structure inspired by gecko feet for rapid sampling and multi-component detection of pesticide residues. The surface-enhanced Raman spectroscopy (G-SERS) platform, based on this gecko-inspired structure, enables the simultaneous detection of the three pesticides using a simple ‘stick-and-lift’ method, as presented in Figure 3. Compared with traditional substrates, G-SERS substrates feature high-density, flexible nano-tentacles that can reach the micro-areas of samples without causing damage. The G-SERS substrate exhibited excellent SERS activity (enhancement factor = 1.2 × 10^7^) and reproducibility (RSD = 5.8%). Direct sampling from the surfaces of cucumbers, apples, and grapes allows the rapid and reliable determination of pesticide residues, including thiram (TMTD), methyl parathion (MPT), malachite green (MG), and their derivatives. Under optimal conditions, the sensitivity of TMTD detection on apple skin is 1.6 ng/cm^2^ (S/N = 3), with a correlation coefficient (R) of 0.9. These findings hold promise for the practical on-site identification of pesticide residues in fruits and vegetables [76,90,91,92,93,94,95,96,97,98,99,100,101,102,103,104,105,106,107,108,109,110,111,112,113,114,115,116,117,118,119,120,121,122,123,124,125,126,127,128,129,130,131,132,133,134,135,136,137,138,139,140,141,142,143,144,145,146,147,148,149,150,151,152,153,154,155,156,157,158,159,160,161,162,163,164,165,166,167,168,169,170,171,172,173,174,175,176,177,178,179,180].

SERS also offers the advantage of multiplexed analysis, enabling the simultaneous detection of multiple pesticides and herbicides in a single measurement. This capability is particularly beneficial for food safety analyses, in which multiple contaminants may be present simultaneously. Using different SERS-active substrates functionalized with specific receptors for different pesticides and herbicides, a comprehensive analysis can be performed in a time-efficient manner, thereby enhancing the throughput of the detection process [91].

One of the most significant advantages of SERS is that it provides non-invasive analysis, which preserves sample integrity during measurement, allowing the reusability of samples for subsequent analyses or confirmation tests. The non-invasive nature of SERS minimizes the need for elaborate sample preparation procedures and diminishes the likelihood of modifying the chemical composition of the sample, thereby ensuring accurate and reliable outcomes in an academic context.

Furthermore, SERS can be coupled with advanced data analysis techniques such as chemometric methods and machine learning algorithms to enhance the accuracy and reliability of pesticide and herbicide detection. These techniques enable the classification and identification of specific pesticides and herbicides based on their unique spectral fingerprints, even in the presence of interference from other compounds. The combination of SERS with data analysis techniques facilitates the development of robust detection models, contributing to the establishment of comprehensive databases for pesticide and herbicide analysis [92].

In conclusion, SERS has demonstrated great potential for the detection of pesticides and herbicides in food samples. Its high sensitivity, multiplexing capabilities, non-destructive analysis, and compatibility with advanced data analysis techniques make it a valuable tool for ensuring food safety and public health. The application of SERS in this context not only enables the rapid and accurate detection of pesticides and herbicides but also contributes to the development of more sustainable agricultural practices and the promotion of consumer confidence in the safety and quality of food products. Ongoing advancements in SERS methodologies, substrate engineering, and analytical approaches will improve their effectiveness and expand their use in detecting and analyzing pesticides and herbicides.

#### 4.1.2. Mycotoxins

Ensuring food safety and public health requires the detection of biological and chemical contaminants in food samples. Among these contaminants, mycotoxins are of particular concern because of their widespread occurrence and adverse health effects, such as carcinogenic, teratogenic, immunosuppressive, and hepatotoxic effects. The toxic secondary metabolites produced by certain fungi require sensitive and reliable analytical methods for their detection [93,94].

For mycotoxin detection in food, SERS has emerged as a powerful technique, combining Raman spectroscopy with plasmonic effects to enhance the Raman signals and allow sensitive detection at low concentrations. This technique offers high sensitivity, selectivity, and multiplexing capability.

The primary advantage of SERS is its high sensitivity. The plasmonic enhancement provided by SERS enables the detection of mycotoxins at trace levels, thus surpassing traditional methods. SERS-active substrates functionalized with specific receptors, such as antibodies or aptamers, selectively capture and detect target mycotoxins, thereby enhancing their sensitivity and specificity [95,96,97]. This allows for the accurate quantification of food samples, aiding in effective risk assessment and mitigation.

SERS offers excellent selectivity, and functionalized SERS-active substrates can specifically bind to target mycotoxins, thus minimizing the interference from other compounds in complex matrices. This selectivity ensures reliable and accurate detection, enabling the differentiation and quantification of different mycotoxin species in food samples [98]. Moreover, SERS facilitates multiplexed analysis, allowing the simultaneous detection of multiple mycotoxins in a single measurement. Using different SERS-active substrates functionalized with specific mycotoxins, comprehensive analysis can be performed efficiently. This capability is particularly valuable because food samples may contain multiple mycotoxin species that require a comprehensive assessment.

**Figure 4:**
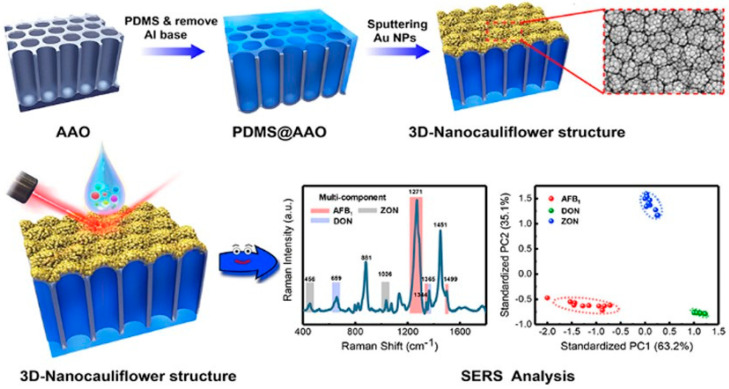
Fabrication process of the cauliflower-inspired 3D SERS substrate. Reprinted with permission from Analytical Chemistry, 2019, 91.6: 3885–3892 [181].

Heyou et al. have introduced a novel three-dimensional SERS substrate inspired by a cauliflower structure. This innovative design aims to detect multiple mycotoxins simultaneously, thereby enhancing food safety measures. The cauliflower-inspired 3D architecture provides an extensive surface area, which improves the sensitivity and detection capabilities of various mycotoxins. By leveraging the natural fractal geometry of cauliflowers, this substrate achieves a high enhancement, enabling the identification of low-concentration contaminants in complex food matrices. The fabrication of 3D SERS substrates is cost effective and scalable. The process involves sputtering gold nanoparticles (Au-NPs) onto a polydimethylsiloxane-coated anodic aluminum oxide (PDMS@AAO) substrate. This method ensures the formation of numerous hot spots, which are critical for enhancing Raman signals, as illustrated in Figure 4.

The effectiveness of this substrate was validated by rigorous testing. The 3D-nanocauliflower SERS substrate demonstrated the ability to detect multiple mycotoxins, including aflatoxin B1 (AFB1), deoxynivalenol (DON), and zearalenone (ZON), with high specificity and sensitivity. The detection limits (LOD) were found to be 1.8 ng/mL for AFB1, 47.7 ng/mL for ZON, and 24.8 ng/mL for DON [99]. The development of this 3D SERS substrate represents a significant advancement in nanomaterial-based detection methods. This not only improves the reliability of mycotoxin detection but also contributes to the broader goal of ensuring food safety through innovative technological solutions. The cost-effectiveness and scalability of this substrate make it suitable for widespread applications in food safety monitoring.

This novel 3D-nanocauliflower SERS substrate offers promise as a tool for the rapid and accurate detection of multiple mycotoxins, enhancing the safety and quality of food products. Continued research and advancement in SERS techniques, substrate design, and receptor functionalization will further enhance their capabilities and broaden their application in mycotoxin analysis, ultimately leading to safer and healthier food products for consumers.

#### 4.1.3. Veterinary Drugs and Antibiotics

The detection of biological and chemical contaminants in food products is crucial to ensure food safety and public health. Among these contaminants, veterinary drugs and antibiotics pose significant concerns because of their potential adverse effects on human health and the development of antibiotic resistance. The accurate and sensitive detection of these compounds is essential for regulatory compliance and the protection of consumer welfare. SERS has emerged as a powerful technique for the detection and analysis of veterinary drugs and antibiotics in food samples [100,101,102,103].

SERS offers several advantages in the detection of veterinary drugs and antibiotics. It syndicates the values of Raman spectroscopy and plasmonic senses to amplify the Raman signals of target analytes, thereby enhancing the sensitivity of detection. This technique enables the identification and quantification of veterinary drugs and antibiotics at trace levels, thereby overcoming the limitations of conventional analytical methods [104].

The plasmonic enhancement provided by SERS allows the detection of these compounds even at low concentrations, enabling accurate quantification and ensuring regulatory compliance. SERS-active substrates functionalized with specific receptors, such as aptamers or antibodies, can selectively capture and detect target veterinary drugs and antibiotics, further enhancing the sensitivity and specificity of analysis.

**Figure 5:**
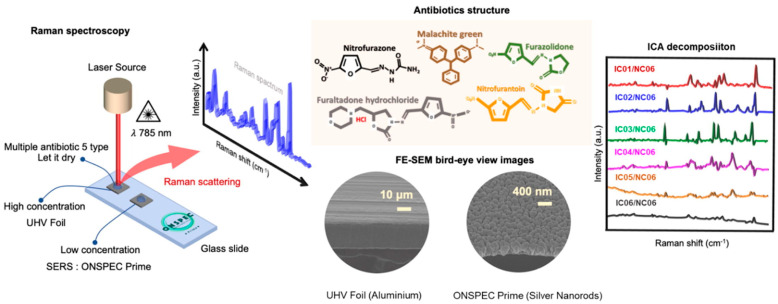
A schematic experimental setup for the detection of antibiotics using Raman spectroscopy and SERS substrates. Reprinted with permission from Spectrochimica Acta Part A: Molecular and Biomolecular Spectroscopy, 2023, 122584 [107].

SERS has further demonstrated outstanding selectivity for the identification of veterinary drugs and antibiotics. Through the utilization of SERS-active substrates equipped with tailored receptors, this method can preferentially attach to desired compounds, thereby reducing potential interference from various components within intricate matrices [105]. This selectivity ensures reliable and accurate detection, enabling differentiation between different veterinary drugs and antibiotics and their quantification in food trials. Furthermore, SERS allows the multiplexed analysis of veterinary drugs and antibiotics. By using different SERS-active substrates functionalized with specific receptors for different compounds, multiple analytes can be simultaneously detected in a single measurement. This capability holds particular value in the analysis of food trials, where the presence of multiple veterinary drugs and antibiotics [106] necessitates thorough screening and assessment, as shown in Figure 5.

SERS has emerged as a highly sensitive technique for the detection and identification of trace amounts of molecules including antibiotics. By utilizing nanostructured substrates, such as silver nanorods (ONSPEC Prime) or aluminum foils (UHV Foil), SERS significantly enhances Raman scattering, enabling the detection of low-concentration analytes that are otherwise undetectable by traditional Raman spectroscopy. In this study, a 785 nm laser source was used to investigate the Raman spectra of various antibiotics deposited on both high- and low-concentration substrates. The morphological characteristics of these substrates, as revealed by FE-SEM imaging, plays a critical role in enhancing the signal intensities. The application of independent component analysis (ICA) allows for the decomposition and identification of spectral data, providing a clear distinction between different antibiotic molecules. This combination of SERS and advanced data analysis techniques is a powerful tool for the rapid, label-free detection of contaminants in complex mixtures, which is essential for applications in environmental monitoring and healthcare diagnostics [107].

SERS is a potent tool for detecting veterinary drugs and antibiotics in food trials. In addition, SERS application in uncovering veterinary drug residues and antibiotic contamination not only enables swift and precise detection but also aids in crafting effective monitoring strategies and risk assessment within the food industry. Ongoing advancements in SERS techniques, substrate design, and receptor functionalization promise to amplify their potential and widen their scope for veterinary drug and antibiotic analyses, ultimately contributing to safer and healthier food products for consumers.

### 4.2. Identification of Foodborne Pathogens

#### 4.2.1. Bacteria (e.g., *Salmonella*, *Escherichia coli*)

The identification of foodborne pathogens, particularly bacteria such as Salmonella [108,109,110] and *Escherichia coli* (*E. coli*) [111,112,113], is of paramount importance to ensure food safety and prevent outbreaks of foodborne illnesses. Traditional bacterial identification methods often involve time-consuming culture-based techniques, which require several days to obtain results. Recently, SERS has gained prominence as a promising method for the rapid and precise identification of bacterial pathogens in food trials. SERS offers several advantages for identifying foodborne bacteria. This method leverages the unique vibrational fingerprints of bacterial molecules to obtain characteristic Raman spectra, which can be used for species-specific identification. This technique relies on the interaction between bacterial molecules and SERS-active substrates, which amplifies the Raman signals and enhances the sensitivity of detection.

It is important to highlight that one of the notable advantages of SERS is its rapidity in foodborne bacterial identification. Compared with traditional culture-based methods, SERS can provide results within minutes, enabling real-time monitoring and prompt intervention in cases of bacterial contamination [114,115,116]. This rapidity is crucial for preventing the spread of foodborne illnesses and ensuring timely implementation of control measures.

**Figure 6:**
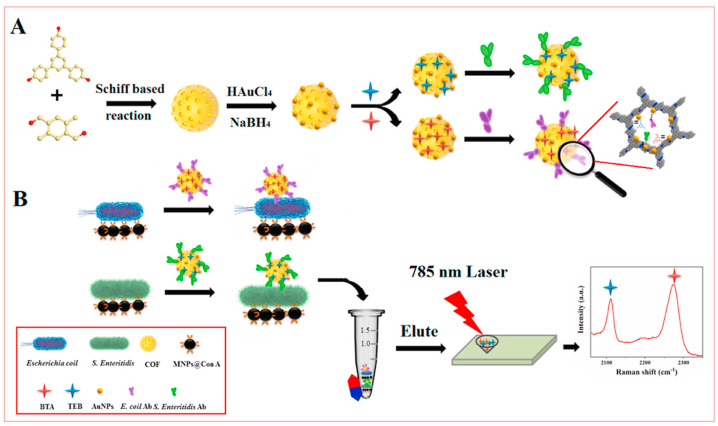
Schematic diagrams show the creation of COF-based Raman tags (**A**) and their application in simultaneous immuno-SERS detection of *E. coli* and *S. enteritidis* (**B**). Reprinted with permission from Talanta, 243 (2022): 123369 [116].

Yan Yang et al. have developed a highly sensitive SERS immunosensor for multiplex detection of foodborne pathogens. This involves the use of biological interference-free Raman tags synthesized within covalent organic framework (COF) TBDP nanocontainers, which load Raman reporters and specific antibodies for target pathogens. Lectin-functionalized magnetic nanoparticles (MNPs@ConA) were employed to capture and isolate multiple pathogens simultaneously by binding to their carbohydrate constituents. Recognition of the target pathogens leads to the formation of MNPs@Con A/pathogen/TBDP@Raman tag sandwich-like composites, which are easily separated using an external magnet, as illustrated in Figure 6. The elution of the collected composites releases a mass of Raman reporters, enabling the simultaneous detection of two different foodborne pathogenic strains with a limit of detection of 10^1^ CFU/mL for each strain. This approach demonstrates a novel application of SERS for pathogenic bacterial detection [117].

SERS also enables the multiplexed identification of foodborne bacteria, preserves sample integrity, and allows for potential sample reuse. By using different SERS-active substrates functionalized with specific receptors for different bacterial species, multiple pathogens can be simultaneously detected in a single measurement. This capability is particularly valuable for the analysis of complex food samples, in which multiple bacterial contaminants may coexist. This minimizes handling and ensures precise and reliable results, thereby benefiting monitoring and control of foodborne pathogens.

In conclusion, SERS has emerged as a powerful tool for the rapid and accurate identification of foodborne pathogens, particularly *Salmonella* and *E. coli*. Its rapidity, sensitivity, specificity, multiplexing capabilities, and non-destructive nature make it a valuable technique for ensuring food safety and preventing the spread of foodborne illnesses. The application of SERS in bacterial identification not only enables timely interventions but also aids in the development of effective control strategies and risk assessment in the food industry. Continued advancements in SERS techniques, substrate design, and receptor functionalization will further enhance their capabilities and broaden their application in the field of foodborne pathogen detection, ultimately leading to safer and healthier food products for consumers.

#### 4.2.2. Viruses (e.g., Norovirus)

Detecting foodborne pathogens, including viruses such as norovirus, plays a critical role in safeguarding food and preventing foodborne disease outbreaks [118,119]. Among the various detection techniques, SERS has emerged as a promising method for the rapid and sensitive identification of viral contaminants in food samples.

SERS presents numerous benefits in the realm of foodborne virus identification, notably for norovirus identification. By leveraging the distinct vibrational properties of viral molecules, SERS can produce distinctive Raman spectra, essentially acting as molecular fingerprints for the precise identification of viral species. This capability facilitates the selective and precise differentiation of various norovirus strains, which is crucial for devising effective control and mitigation measures [120,121]. SERS also enables the multiplexed identification of foodborne viruses, including norovirus. By utilizing SERS-active substrates functionalized with specific receptors such as antibodies or aptamers, multiple viral strains can be simultaneously detected in a single analysis. This capability is particularly valuable for the analysis of complex food matrices in which multiple viral contaminants may coexist [122].

SERS offers a significant advantage for viral identification because of its rapidity. This is in contrast with traditional detection methods for norovirus, which often involve time-consuming and labor-intensive techniques such as polymerase chain reaction (PCR) or enzyme-linked immunosorbent assay (ELISA). SERS can provide rapid results within minutes, enabling real-time monitoring and timely intervention in cases of viral contamination. Moreover, SERS provides outstanding sensitivity and specificity for the detection of norovirus. Signal amplification enabled by SERS-active substrates enables the detection of viral particles even at low concentrations, surpassing the limitations of conventional techniques. SERS can distinguish norovirus from other viral contaminants or nonpathogenic particles, thereby minimizing false-positive or false-negative results [123].

**Figure 7:**
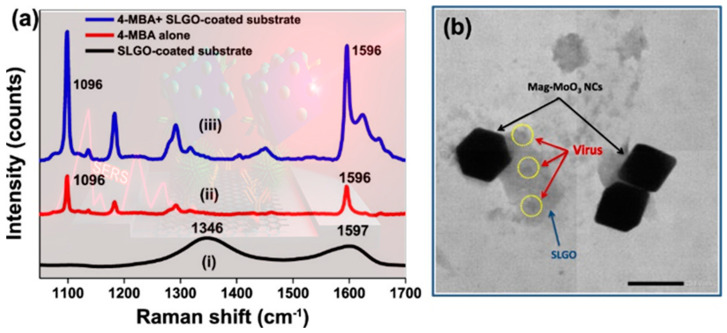
(**a**) Raman spectra of three samples: (i) SLGO capture substrate, (ii) 4-MBA on an untreated glass slide, and (iii) SLGO-4MBA substrate. (**b**) TEM image illustrating the sandwich-type immunocomplex consisting of mag-MoO_3_/NoV-LPs/4-MBA-antibody-SLGO. Reprinted with permission from ACS Applied Materials and Interfaces (2020), 12(39):43522-43534 [123].

Ojodomo J. Achadu et al. 2020, created a novel biosensing system using graphene-mediated surface-enhanced Raman scattering alongside plasmonic/magnetic molybdenum trioxide nanocubes (mag-MoO_3_ NCs) as shown in the TEM image at Figure 7b. This system was designed for norovirus detection using a dual SERS nanotag/substrate setup. The biosensor was tested with NoV-like particles to evaluate its performance, revealing a wide linear detection range spanning from 10 fg/mL to 100 ng/mL and a highly sensitive limit of detection of approximately 5.2 fg/mL, as presented in Figure 7a. Additionally, the biosensor demonstrates practical usability for the detection of clinical NoV subtypes in human fecal samples, achieving an LOD of approximately 60 RNA copies/mL. Importantly, this LOD is approximately 103 times lower than that of a commercial enzyme-linked immunosorbent assay kit for NoV [124,125].

In summary, SERS has become a compelling tool for the rapid and sensitive detection of foodborne viruses, including norovirus. Its speed, sensitivity, specificity, multiplexing capacity, and non-destructive nature make it invaluable for guaranteeing food safety and halting foodborne disease outbreaks, as presented in Table 2. The utilization of SERS in viral identification not only allows for timely responses but also contributes to the formulation of effective control measures and risk assessments in the food industry. Ongoing advancements in SERS techniques, substrate design, and receptor functionalization will further augment the capabilities of SERS and broaden its applications in foodborne pathogen detection, ultimately ensuring safer and healthier food products for consumers.

### 4.3. Authentication and Quality Control of Food Products

#### 4.3.1. Adulteration Detection (e.g., Food Fraud)

The authentication and quality control of food products are critical for ensuring consumer safety and maintaining the integrity of the food industry. Among the various techniques available, SERS has emerged as a powerful tool for the detection and authentication of food adulteration, which is commonly known as food fraud. Food fraud refers to the intentional misrepresentation or adulteration of food products for economic gain, which can have serious implications for consumer health and trust in the food supply chain. SERS offers several advantages in the detection of adulterants, enabling the accurate and reliable authentication of food products [149].

SERS presents a notable advantage for the authentication of food products through molecular fingerprinting. Through analysis of the distinct Raman spectra produced by food samples, SERS can discern specific chemical compounds or adulterants that serve as indicators of food fraud. This molecular fingerprinting approach allows for the detection of adulterants at trace levels, surpassing the limits of conventional methods.

SERS also offers excellent sensitivity and selectivity for detecting food adulteration. The signal enhancement provided by SERS-active substrates enables the detection of adulterants even at low concentrations, ensuring reliable results. Furthermore, SERS can distinguish between different types of adulterants, including unauthorized additives, substitution of inferior ingredients, and dilution of high-value products [150].

Moreover, SERS allows for rapid and non-destructive analysis of food products during the authentication process. Traditional methods of detecting food fraud often involve time-consuming sample preparation or destructive techniques. By contrast, SERS enables real-time monitoring and analysis of food samples without the need for extensive sample preparation or alteration, thereby preserving the integrity of the product. Furthermore, SERS can be coupled with chemometric analysis, such as multivariate data analysis, or pattern recognition algorithms to enhance the accuracy and reliability of food authentication. By combining the spectral information obtained from SERS with statistical models, robust authentication models that can classify and quantify different types of food adulteration can be developed [151].

In conclusion, SERS has emerged as a powerful tool for the authentication and quality control of food products, particularly in the detection of food adulteration and fraud. Its ability to provide molecular fingerprinting, sensitivity, selectivity, rapidity, non-destructive analysis, and compatibility with chemometric analysis makes it highly suitable for addressing challenges associated with food fraud. The application of SERS in food authentication not only helps protect consumer health but also strengthens trust and transparency in the food industry. Continued research and advancements in SERS techniques and data analyses will further enhance their capabilities and contribute to effective quality control and regulation in the food sector.

#### 4.3.2. Origin and Authenticity Verification

Verification of the origin and authenticity of food products plays a crucial role in ensuring consumer safety, protecting the integrity of the food supply chain, and preventing fraudulent practices. SERS has emerged as a powerful scientific tool for the rapid and accurate verification of the origin and authenticity of food products.

SERS offers several advantages in terms of origin and authenticity verification. One of the key advantages of this method is its ability to provide molecular fingerprints, allowing the identification and differentiation of food products based on their unique chemical composition. By analyzing the Raman spectra obtained from food samples, SERS can be used to detect and characterize the specific molecular markers that are indicative of the origin or authenticity of the product.

The sensitivity and selectivity of SERS enables the detection of trace levels of chemical compounds, allowing for the identification of even minor variations in food products. This is particularly important for verifying the geographic origin of agricultural products and authenticity of high-value food items. SERS can detect and quantify specific markers such as natural pigments or bioactive compounds, which are characteristic of specific regions or production methods, providing valuable information about the authenticity of the product.

Furthermore, SERS enables the rapid and non-destructive analysis of food samples, minimizing the need for extensive sample preparation or alteration. This non-destructive nature of SERS is advantageous for preserving the integrity and value of food products being analyzed. By avoiding destructive techniques such as sample extraction or chemical modification, SERS ensures that the food product remains intact and can be safely consumed or further processed after analysis.

In addition to molecular fingerprinting, SERS can be combined with statistical models and chemometric analyses to develop robust classification and prediction models for verifying its origin and authenticity. By creating spectral databases and utilizing advanced data analysis techniques, SERS can provide objective and reliable results for the identification of food product origins or the detection of fraudulent practices [152].

The application of SERS for origin and authenticity verification extends to a wide range of food products, including fruits, vegetables, meat, fish, wine, and dairy products. Its versatility and compatibility with various sample matrices make it a valuable tool for addressing the challenges associated with product labeling, geographic origin claims, and counterfeit products.

SERS has emerged as a powerful scientific tool for verifying the origins and authenticity of food products. Its ability to provide molecular fingerprinting, sensitivity, selectivity, rapidity, non-destructive analysis, and compatibility with statistical models makes it highly suitable for addressing the complexities of origin and authenticity verification. By employing SERS techniques, food regulatory authorities, producers, and consumers can ensure the integrity and authenticity of food products, thereby promoting consumer trust, safeguarding public health, and enhancing transparency in the food industry. Further advancements in SERS technology and data analysis will continue to expand their application and contribute to the advancement of origin and authenticity verification in the food sector.

### 4.4. Analysis of Food Packaging Materials for Safety Assessment

The analysis of food packaging materials for safety assessment is of utmost importance to ensure the quality and integrity of packaged food products. The choice of packaging material can have a significant impact on the safety and shelf life of food as well as the potential migration of harmful substances into the food matrix. SERS has emerged as a valuable tool for the analysis of food packaging materials, offering rapid and reliable characterization of their chemical composition and identification of potential contaminants [153].

SERS provides several advantages for the analysis of food packaging materials. One of the key advantages of this method is its ability to detect and identify a wide range of chemical compounds, including volatile organic compounds (VOCs), additives, monomers, and degradation products, that may be present in packaging materials. By analyzing the Raman spectra obtained from the packaging materials, SERS enables the identification of specific molecular markers, enabling the assessment of their safety and compliance with regulatory standards [154].

The sensitivity and selectivity of SERS make it suitable for the detection and quantification of trace levels of contaminants that may migrate from packaging materials to food. These contaminants include potential endocrine disruptors, heavy metals, plasticizers, and other harmful substances that pose risks to human health. SERS allows the identification and quantification of these contaminants, even at low concentrations, thereby ensuring the safety and compliance of packaging materials.

Furthermore, the non-destructive nature of SERS analysis is advantageous for evaluating packaging materials. This enables the assessment of both the inner and outer surfaces of packaging without causing damage or alteration of the material. This non-destructive analysis allows for the preservation of packaging integrity, ensuring that the material can be further evaluated for its barrier properties, mechanical strength, and overall performance. SERS can also be combined with chemometric analysis and statistical modelling to develop predictive models for the safety assessment of food packaging materials. By creating spectral libraries and utilizing advanced data analysis techniques, SERS can provide objective and reliable results for the identification of potential contaminants and the evaluation of their impact on food safety.

The application of SERS in the analysis of food packaging materials extends to various packaging types, such as plastics, glass, metals, and paper-based materials. Its versatility allows for the assessment of different packaging components, including coatings, adhesives, printing inks, and laminated layers. This comprehensive analysis provides valuable insights into the safety and suitability of packaging materials for specific food applications [155,156].

In conclusion, SERS offers a powerful scientific tool for the analysis of food packaging materials, enabling the rapid and reliable assessment of their chemical composition, identification of potential contaminants, and evaluation of their safety for food contact. Its sensitivity, selectivity, non-destructive nature, and compatibility with statistical models make it highly suitable for addressing the complexities of food packaging safety assessments. By employing SERS techniques, regulatory authorities, packaging manufacturers, and food producers can ensure the safety, quality, and compliance of food packaging materials, thereby safeguarding public health and promoting consumer confidence in food supply chains. Further advancements in SERS technology and data analysis approaches will continue to enhance their application in the analysis of food packaging materials for safety assessment.

## 5. Challenges and Future Perspectives

### 5.1. Integration of Big Data Analysis and Usage of Artificial Intelligence (AI)

The field of SERS for food safety detection is experiencing rapid advancements; nevertheless, several challenges persist that must be addressed in order to fully realize its potential. Among these challenges is the integration of big data analysis and the utilization of artificial intelligence (AI) to enhance SERS capabilities.

Big data analysis holds immense promise in transforming SERS into a robust and reliable tool for food safety detection. The vast amount of data generated by SERS experiments, encompassing a wide range of spectral information, can be overwhelming and difficult to interpret without advanced analytical tools. Big-data techniques can process and analyze large datasets to identify patterns, correlations, and trends that may not be immediately apparent. This integration can enhance the accuracy and reliability of SERS for contaminant detection [157]. For instance, big data analysis can facilitate the development of comprehensive spectral libraries for various mycotoxins and other contaminants.

The combination of SERS and deep learning models, specifically one-dimensional and two-dimensional convolutional neural networks (1D CNNs and 2D CNNs, respectively), provides an ultrasensitive and effective method for detecting trace levels of zearalenone (ZEN) in corn oil. The study demonstrated that these deep learning models outperformed traditional regression models (PLSR, GPR, RFR) in terms of prediction performance. The proposed method showed high accuracy, stability, and sensitivity, with limits of detection (LOD) of 6.81 × 10^4^ μg/mL for 1D CNN and 7.24 × 10^4^ μg/mL for 2D CNN, which are lower than the maximum residue limits set by China and the European Union [158].

These libraries can then be used as reference databases to identify unknown samples quickly and accurately. Additionally, big data techniques can help optimize experimental conditions by analyzing historical data to determine the most effective parameters for SERS detection. The application of big data in SERS also extends to the quality control and validation of data. By continuously monitoring and analyzing data streams, big data tools can identify anomalies and outliers, ensuring that the data collected are consistent and reliable. This is particularly important in fields in which precision and accuracy are critical [159,160].

On the other hand, AI has the potential to revolutionize the application of SERS in food safety detection. AI algorithms, particularly machine learning (ML) and deep learning (DL) can be trained to recognize complex spectral patterns and make accurate predictions based on large datasets. The integration of AI into SERS can enhance both the speed and accuracy of mycotoxin detection [161,162].

Machine learning algorithms can be used to develop predictive models that correlate specific spectral features with the presence and concentration of contaminants. These models can be continuously refined and improved as more data are collected, leading to more accurate and reliable detection methods. Deep learning, with its ability to handle large and complex datasets, can further enhance the ability of these models to identify subtle spectral differences that may indicate contamination.

Moreover, AI can facilitate SERS detection automation. Automated systems equipped with AI can perform real-time analyses of SERS spectra, providing immediate feedback regarding the presence of contaminants. This can significantly reduce the time required for analysis and enable rapid decision making, which is crucial for food safety applications [163,164].

The future of SERS for food safety detection lies in the seamless integration of big data with AI. Developing standardized protocols for data collection and sharing is essential to harness the full potential of big data and AI. Collaborative efforts between researchers, industry stakeholders, and regulatory bodies can lead to the creation of comprehensive spectral databases and advanced analytical tools. Additionally, the development of user-friendly software and interfaces for big data and AI applications in SERS is crucial. These tools should be accessible to non-experts to enable the wider adoption of SERS technology in food safety monitoring [165,166].

Researchers should focus on improving the sensitivity and specificity of SERS substrates. Combining advanced nanomaterials with AI-driven optimization can lead to the creation of highly efficient and reliable SERS platforms. Furthermore, exploring the potential of portable and handheld SERS devices equipped with AI capabilities can open new avenues for on-site and real-time food safety monitoring [167,168,169]. The integration of big data analysis and AI into SERS represents a significant advancement in the field of food safety detection. These technologies have the potential to enhance the sensitivity, accuracy, and efficiency of SERS, thereby rendering it a robust tool for ensuring the safety and quality of food products. Continued research and interdisciplinary collaboration will be essential to overcome the current challenges and fully realize the potential of SERS in this critical application.

### 5.2. Standardization and Validation of SERS Techniques

Ensuring the reliability, reproducibility, and comparability of SERS data requires rigorous standardization and validation. Standardization involves creating consistent protocols and performance criteria for sample preparation, instrumentation, data acquisition, analysis, and reporting. Key aspects include uniform sample preparation (substrate selection, deposition methods, and analyte concentrations) and standardized instrumental parameters (laser wavelength, power, and acquisition time). In contrast, validation assesses the performance characteristics of SERS techniques, such as the sensitivity, selectivity, accuracy, precision, and limit of detection. This process typically involves the use of reference materials and statistical analysis to ensure reliability and accuracy. Inter-laboratory studies and collaborative efforts are crucial, involving multiple laboratories working together to evaluate methods, identify sources of variability, and establish best practices.

The development and dissemination of standardized reference materials, such as SERS substrates with known properties, further support these efforts. These materials facilitate calibration, quality control, and traceability, thereby ensuring accuracy and comparability across studies. In summary, standardization and validation of SERS techniques are essential for generating reliable and comparable data. The scientific community can enhance the credibility and applicability of SERS in various fields by establishing consistent protocols and performance criteria and by utilizing inter-laboratory studies and reference materials [170].

### 5.3. Regulatory Approvals, Market Readiness, and Barriers to Commercialization

The adoption of SERS in sectors such as food safety, pharmaceuticals, and clinical diagnostics is contingent upon compliance with stringent regulatory frameworks established by organizations such as the Food and Drug Administration (FDA), the European Medicines Agency (EMA), and the International Organization for Standardization (ISO). Despite its significant potential in laboratory settings, obtaining widespread regulatory approval for SERS remains challenging. This is largely because of the requirement for rigorous validation studies that must demonstrate the consistency, reproducibility, and specificity of the technology across a wide range of applications [171].

Regarding market readiness, SERS technology has undergone considerable advancements, with commercial SERS substrates and devices now available for research purposes. However, their broader adoption faces challenges related to scalability, cost efficiency, and usability. The development of more affordable and reproducible SERS substrates is essential to facilitate their integration into routine commercial applications, particularly in resource-constrained settings. In response to these demands, companies are increasingly investing in innovations aimed at enhancing the user-friendliness of SERS devices, enabling non-specialists to conduct analyses without the need for extensive training [172].

However, several barriers to its commercialization remain. A key challenge is the high cost associated with the production of SERS substrates that maintain consistent quality and performance. This factor may limit the technology’s uptake in budget-sensitive sectors such as public health and environmental monitoring. Moreover, the lack of standardized SERS protocols across various applications poses an additional hurdle, as standardization is crucial for ensuring the comparability of results and fostering broader acceptance. Furthermore, the complexity involved in interpreting SERS data, which often necessitates sophisticated instrumentation and expertise, can limit its accessibility to non-specialist users.

Several strategies have been proposed to address these issues. First, enhanced collaboration between academic researchers, industry stakeholders, and regulatory bodies is necessary to establish standardized protocols and validation frameworks. Second, continued investment in scalable and cost-effective production techniques for SERS substrates, potentially leveraging advancements in nanofabrication technologies, is pivotal for improving the commercial viability of SERS. Finally, automating SERS data interpretation through software innovations that simplify and standardize the analysis will make the technology more accessible to a broader range of users without requiring specialized expertise. By overcoming these regulatory, market, and technical challenges, we believe that SERS has significant potential to become a widely adopted analytical tool in numerous industries.

### 5.4. Sample Preparation and Matrix Effects

Sample preparation and matrix effects are critical considerations in the application of SERS for the analysis of complex samples to obtain reliable and accurate SERS measurements and significantly influence SERS signal and detection limits. This process may include sample homogenization, filtration, extraction, concentration, and purification steps, depending on the nature of the sample and analytes of interest. Appropriate sample preparation is crucial for minimizing interference, removing impurities, and enhancing the accessibility of analytes to the SERS-active substrates [173]. Matrix effects refer to the interactions between the sample matrix components and the SERS substrates, which can affect the intensity and reproducibility of the SERS signal. Matrix effects can arise from various factors, such as the chemical composition, pH, viscosity, surface properties, and optical properties of the sample matrix. These effects can lead to signal enhancement or suppression, spectral distortions, or variations in the detection limits, potentially hindering the accuracy and reliability of SERS analysis.

Several strategies can be employed during sample preparation to mitigate the matrix effects. One approach is to selectively separate or extract the target analytes from the matrix using techniques such as solid phase extraction, liquid–liquid extraction, or immunoaffinity chromatography. By isolating the analytes from the interfering matrix components, the SERS signal can be more accurately attributed to the analytes of interest [76].

Another strategy involves using appropriate sample dilution or concentration techniques to optimize the analyte-to-matrix ratio for SERS analysis. Dilution can help reduce the matrix effects by minimizing the concentration of interfering components, whereas concentration techniques such as evaporation or solid phase microextraction can enhance the SERS signal by enriching the analytes. Moreover, the choice of SERS substrate can also influence the extent of the matrix effects. Tailoring the substrate properties, such as surface chemistry, morphology, and size, can help optimize the interaction between the analytes and substrate, minimize matrix effects, and enhance the sensitivity and selectivity of SERS measurements [174,175]. Understanding and characterizing the matrix effects associated with different sample matrices are essential for the accurate interpretation and quantification of SERS data. Calibration curves using matrix-matched standards can help to compensate for matrix effects and improve the accuracy of analyte quantification. Additionally, statistical approaches such as chemometric and multivariate data analyses can be employed to account for matrix effects and extract meaningful information from complex SERS spectra.

In summary, proper sample preparation techniques can enhance the reliability and accuracy of SERS measurements by minimizing interference and optimizing the analyte accessibility. Understanding and mitigating matrix effects through appropriate sample treatment, substrate selection, and data analysis strategies is essential for obtaining meaningful and reproducible results in SERS analysis of diverse sample matrices.

### 5.5. Integration of SERS with Portable and User-Friendly Devices

The incorporation of SERS into portable and user-friendly devices represents a significant breakthrough in this field, enabling on-site and point-of-care applications. The miniaturization and creation of portable SERS systems have facilitated the real-time and rapid detection of analytes in various fields, including food safety, ecological monitoring, biological diagnostics, and forensics [176]. Portable SERS devices are designed to be small, lightweight, and simple to operate, enabling non-experts to perform measurements in diverse settings. These devices typically consist of a laser source, SERS-active substrate, Raman signal collection system, and user interface for data acquisition and analysis. Integration of these components into a single handheld device offers portability, convenience, and immediate data interpretation.

The use of mobile SERS devices in food safety has markedly enhanced the detection of contaminants, allergens, and adulterants. These devices facilitate swift screening of food samples across the entire supply chain, ranging from production to distribution and consumption. The capability of real-time analysis provided by portable SERS devices enables immediate decision making, thereby ensuring the safety and quality of food products. In addition, the combination of SERS with user-friendly devices has expanded the accessibility of this technique to non-specialized users, such as field technicians, healthcare professionals, and law enforcement personnel. The simplified operation and intuitive user interfaces of these devices render them suitable for point-of-care diagnostics, environmental monitoring in remote areas, and on-site forensics analysis. Advances in portable SERS devices have been accompanied by the development of data-processing and analysis algorithms. These devices often incorporate smart algorithms and software tools that enable real-time data analysis, pattern recognition, and automated identification of the target analytes. The integration of SERS with intelligent data processing enhances the speed, accuracy, and reliability of analysis, making it more accessible and user-friendly.

The combination of SERS technology with portable and user-friendly devices has enabled this highly effective spectroscopic technique to extend beyond laboratory applications. This has facilitated real-time and on-site monitoring, reduced dependence on centralized analytical facilities, and provided quick and precise results for a diverse range of applications. It is anticipated that ongoing improvements in miniaturization, user interface design, and data processing algorithms will further enhance the functionality and accessibility of portable SERS devices, thereby creating new opportunities for point-of-need analyses and personalized diagnostics.

### 5.6. Exploration of New SERS Substrates and Enhancement Strategies

The investigation of novel SERS substrates and enhancement strategies have been the focus of extensive research in recent years, driven by a desire to improve the sensitivity, reproducibility, and versatility of SERS-based analyses. SERS substrates are crucial for enhancing the Raman signals of analytes, enabling the detection of ultra-low concentrations and facilitating the identification of complex molecular structures. Researchers have actively explored various types of SERS substrates including metallic nanoparticles, nanostructures, and hybrid materials. Metallic nanoparticles, such as gold and silver nanoparticles, have shown exceptional SERS enhancements owing to their unique plasmonic properties. These nanoparticles can be engineered into different shapes and sizes, such as nanospheres, nanorods, and nanostars, to achieve tailored enhancements [177,178]. Moreover, novel nanomaterials, such as graphene, carbon nanotubes, and metal–organic frameworks, have been explored as alternative SERS substrates, offering distinct advantages, such as large surface areas, high chemical stability, and tunable optical properties.

In parallel, researchers have investigated various enhancement strategies to improve the sensitivity and reproducibility of SERS measurements. These strategies aim to maximize electromagnetic enhancement and minimize unwanted background signals. Furthermore, surface engineering techniques, such as surface roughening, surface functionalization, and molecular imprinting, have been employed to optimize the interactions between analyte molecules and SERS substrates. These approaches enhance the adsorption of target molecules onto the substrate surface, promote the formation of hotspots, and minimize the influence of interfering species, thereby improving the sensitivity and selectivity of the SERS measurements.

The investigation of innovative SERS substrates and enhancement tactics has considerable potential for improving the SERS-centric analysis. By extending the limits of sensitivity, reproducibility, and versatility, researchers are creating new opportunities for the utilization of SERS in a wide range of fields including chemical sensing, biomedical diagnostics, eco-friendly monitoring, and food safety evaluation. It is anticipated that ongoing work in this domain will result in the development of novel SERS platforms with enhanced performance and broader applicability, thus contributing to the progress in analytical sciences and technologies [179].

## 6. Conclusions

### 6.1. Recap of the Importance and Potential of SERS in Rapid Food Safety Detection

SERS has emerged as a robust solution to address food safety concerns. Owing to its remarkable sensitivity and selectivity, SERS enables precise identification of minute analytes in food samples. Notably, SERS boasts multiplexing capabilities, facilitating the concurrent detection of multiple contaminants in a single assessment, thereby conserving both time and resources. The non-destructive nature of SERS is invaluable because it safeguards the sample integrity for subsequent testing or storage purposes. Moreover, the deployment of portable SERS devices for on-site, real-time monitoring elevates food safety protocols and facilitates prompt decision making. In addition to detecting contamination, SERS is useful for verifying food authenticity, detecting adulteration, and countering fraud. Its application extends to verifying the origin and authenticity of food products. Nonetheless, achieving consistent results depends on the standardization and validation of SERS techniques. Effective utilization requires meticulous consideration of sample preparation techniques and matrix effects to optimize the SERS analysis. The integration of SERS with user-friendly portable devices enhances accessibility and practicality. To fully harness SERS’s potential in rapid food safety detection, continual research, collaborative efforts, and technological advancements remain imperative.

### 6.2. Final Thoughts on the Future Prospects and Impact of SERS in Ensuring Food Safety

The use of SERS for ensuring food safety is highly promising and has the potential to revolutionize the field of food analysis and monitoring. The unique capabilities of SERS, such as its high sensitivity, selectivity, and multiplexing ability, make it a valuable tool for the rapid and accurate detection of various contaminants and analytes in food matrices. A significant advantage of SERS is its ability to precisely detect minute quantities of contaminants, including chemical pollutants, toxins, and pathogens. This heightened sensitivity facilitates early identification and intervention, consequently mitigating the hazards associated with tainted food items and diminishing the likelihood of foodborne illness.

Moreover, SERS offers the advantage of non-destructive analysis, allowing for the preservation of sample integrity and the possibility of subsequent analysis. This is particularly important when assessing the quality and authenticity of food products as it allows for the identification of adulterants, contaminants, and fraudulent practices without compromising the sample. The future impact of SERS in ensuring food safety lies not only in its analytical capabilities, but also in its potential for integration with portable and user-friendly devices. This opens up new possibilities for on-site and real-time monitoring, enabling the rapid and efficient screening of food products at various stages of the supply chain. By providing immediate feedback, SERS can facilitate prompt decision making, interventions, and quality control measures to ensure the safety and integrity of the food supply.

However, further research and development are needed to address some of the challenges associated with SERS, such as the standardization and validation of techniques, optimization of sample preparation methods, and overcoming matrix effects. Collaborative efforts among researchers, regulators, and industry stakeholders are crucial to address these challenges and establish robust protocols and guidelines for the widespread implementation of SERS in food safety applications. In summary, SERS is a promising technique for ensuring food safety, with the potential to significantly enhance the detection, monitoring, and control of contaminants in food products. By leveraging its unique analytical capabilities and advancing its integration with portable devices, SERS has the potential to revolutionize food safety practices, minimize public health risks, and contribute to the overall improvement of food quality and security.

### 6.3. Highlighting the Need for Continued Research and Development in This Field

Although SERS has shown tremendous potential in ensuring food safety, it is important to emphasize the need for continued research and development in this field. Although SERS offers numerous advantages, several challenges and areas of research require further investigation.

One of the key areas that warrant attention is the standardization and validation of SERS techniques. Establishing standardized protocols, reference materials, and quality control measures is essential to ensure the reliability and reproducibility of SERS results. This will enable comparison of data across different laboratories and facilitate the adoption of SERS as a routine analytical tool for food safety testing.

Another critical aspect is the optimization of sample preparation methods to minimize matrix effects. Food matrices can contain complex components that interfere with Raman signals, thereby affecting the accuracy and sensitivity of analysis. Developing efficient and robust sample preparation techniques that can effectively remove interference and enhance signal-to-noise ratios is crucial for reliable and precise SERS analyses in food safety applications.

Furthermore, the exploration of new SERS substrates and enhancement strategies is vital to expand the capabilities of this technique. Novel substrates with enhanced sensitivity, stability, and reproducibility can improve detection limits and broaden the range of analytes that can be detected using SERS. Additionally, exploring new enhancement strategies such as plasmonic nanostructures and functionalized surfaces can further amplify Raman signals, enabling even greater sensitivity and selectivity in food safety analysis. In addition to technical advancements, cooperation between researchers, regulators, and industry stakeholders is paramount. By working together, it is possible to establish harmonized guidelines, validate SERS methods against established reference methods, and address regulatory requirements for the implementation of SERS in food safety testing. Collaborations will also enable the exchange of expertise, dissemination of effective strategies, and recognition of new challenges and prospects in this swiftly advancing domain. In conclusion, although SERS has demonstrated immense potential for ensuring food safety, continued research and development efforts are essential for overcoming technical challenges, improving analytical performance, and establishing robust protocols. By addressing these issues, SERS can be a powerful tool for the rapid and reliable detection of contaminants, contributing to enhanced food safety practices and public health protection.

## Figures and Tables

**Table 2:** Comprehensive overview of SERS applications, highlighting the materials, methods, and sensitivities achieved in various fields.

SERS Application	Case Study	Nanomaterials/Substrates Used	Preparation Method	Sensitivity Achieved	Ref.
Pesticide Detection	Detection of organophosphorus pesticides	Ag/Au bimetallic nanoparticles	Acetylcholinesterase SERS biosensor	In situ detection of pesticide residues	[126,127]
	Detection of thiram in food samples	Sliver nanoparticles AgNP-based SERS	Chemical reduction of silver salts, followed by functionalization	Detects at sub-femtomolar concentrations, reaching single-molecule levels.	[128,129]
	Detection of malachite green in water	Hydrophobic SiNRs with Au nanoparticles	Plasma etching and magnetron sputtering	Detection at 1 ng/mL	[130]
Mycotoxin Detection	Detection of aflatoxin B1 in corn	Novel nanostructured materials	Bottom-up and top-down nanofabrication	Enhanced specificity and reproducibility	[131]
	Detection of aflatoxin in food matrices (Corn)	Gold nanoparticles (AuNPs) using AuNP-based SERS	Citrate reduction of HAuCl4, functionalized with specific ligands	Picomolar detection limits for aflatoxin in corn samples	[132,133]
Foodborne Pathogen Detection	Detection of bacterial pathogens in food	Gold nanoparticles	Machine learning-assisted SERS	Rapid, sensitive detection of bacterial pathogens	[134]
	Detection of *E. coli* in milk	Silver-coated nanoporous silicon	Indirect immunoassay with 4-ATP/Ag-pSi	3 CFU/mL detection limit	[135]
	Detection of salmonella in food using magnetic-SERS	Magnetic nanoparticles coated with Ag or Au	Coating magnetic nanoparticles with Ag/Au via chemical deposition	Detection limit as low as a few colony-forming units (CFU)	[113]
Heavy Metal Detection and Environmental Monitoring	Detection of mercury ions	Upconversion nanoparticles (UCNPs)	Lanthanide ion-doped	Rapid, cost-effective detection	[136]
		Gold nanoparticles (AuNPs)	Citrate reduction followed by ligand functionalization	Sub-nanomolar detection limit for Hg2+ ions	[137]
	Detection of PAHs	Pd@Au nanocomposite	Ion irradiation on thin films	5 μM for Methylene Blue	[138]
Chemical Warfare Agent Detection	Detection of methyl salicylate	Gold nanoparticle nanofibers	SERS substrate preparation for chemical warfare simulants	Picomolar detection	[139]
	Detection of organophosphates (e.g., Sarin)	Silver-coated silicon nanowires (Ag-SiNWs)	Vapor-phase deposition of silver onto silicon nanowires	Picomolar detection of organophosphates	[126,140]
Drug Detection	Detection of illegal drugs (e.g., cocaine) using AgNPs	Silver nanoparticles (AgNPs)	Chemical reduction followed by colloidal dispersion	Nanomolar sensitivity for cocaine detection	[141]
Detection of Antibiotics in Food	Detection of ampicillin in milk	3D plasmonic cavity-in-cavity SERS platform	Machine learning-driven, truncated concave nanocubes	A detection limit of 0.1 ppm	[142]
	Detection of ampicillin and nitrofurantoin	Au nanoparticles/graphene oxide hybrid	In situ reduction method	Detection limits as low as 0.01 ng/mL	[143]
Biological Toxin Detection	Detection of aflatoxin B1 in food matrices	Au@Ag nanoparticles	Uniform synthesis for detection	Detection limit of 10^−8^ M	[144]
	Detection of Ricin toxin using AgNP-based SERS	Silver nanoparticles (AgNPs)	Chemical reduction and surface modification	A detection limit as low as 0.32 fM	[145]
Pharmaceuticals Monitoring	Detection of active pharmaceutical ingredients (APIs)	Gold-coated polystyrene nanospheres (Au@PS)	Layer-by-layer assembly of polystyrene beads coated with gold	Nanomolar detection of APIs in pharmaceutical samples	[146]
Food Quality Control	Detection of 4-mercaptopyridine	Au-nanoparticle-decorated cotton swabs (CS-Au NP)	Dropwise addition of gold colloid on cotton fibers	Detection at 1 × 10^−8^ M	[147]
Pesticide Residue Analysis	Analysis of pesticide residues in fruits and detection limit for crystal violet	ZnO nanorods decorated with Ag nanoflowers	Hybrid substrate with “hotspots” engineering	A detection limit of 10⁻^13^ M	[148]
